# Uncovering stromal cell fate genes and a novel risk stratification in UCEC by integrating single-cell RNA sequencing and multi-omics analysis

**DOI:** 10.1016/j.gendis.2025.101743

**Published:** 2025-06-27

**Authors:** Rulin Zhang, Haonan Ma, Yiting Yang, Siang Lv, Xin Guan, Bingnan Lu, Yuntao Yao, Runzhi Huang, Yifan Liu, Yanhua Du, Jun Wu

**Affiliations:** a Department of Laboratory Medicine, Jiading Branch of Shanghai General Hospital, Shanghai Jiao Tong University School of Medicine, Shanghai 201803, China; b Department of Laboratory Medicine, Shanghai General Hospital, Shanghai Jiao Tong University School of Medicine, Shanghai 200080, China; c Department of Ophthalmology, Xinhua Hospital Affiliated to Shanghai Jiao Tong University School of Medicine, Shanghai 200092, China; d Xinhua Hospital Affiliated to Shanghai Jiao Tong University School of Medicine, Shanghai 200092, China; e Department of Pathology, The Affiliated Hospital of Youjiang Medical University for Nationalities, Baise, Guangxi 533000, China; f Key Laboratory of Tumor Molecular Pathology of Guangxi Higher Education Institutes, Baise, Guangxi 533000, China; g Department of Urology, Xinhua Hospital Affiliated to Shanghai Jiao Tong University School of Medicine, Shanghai 200092, China; h Department of Burn Surgery, The First Affiliated Hospital of Naval Medical University, Shanghai 200433, China; i Department of Gynecology, Obstetrics and Gynecology Hospital of Fudan University, Shanghai 200011, China

Endometrial cancer, one of the most prevalent gynecological malignancies, has been steadily increasing in incidence, with 417,000 new cases and 97,000 deaths reported worldwide in 2020.^1^ Its classification is crucial for effective management. Endometrial cancer is a highly heterogeneous tumor, exhibiting significant variation in clinical outcomes even within defined grades and tissue types.^2^ An evaluation of the five leading risk stratification systems for endometrial cancer revealed that none achieved high accuracy in predicting recurrence or metastasis, resulting in substantial treatment variations for the same patient based on differing criteria.^3^ Stromal stem cells were regarded as one of the primary contributors to the origin of endometrial cancer.^4^ These cells could initiate a vicious cycle of tumor development through extensive crosstalk with tumor cells, suggesting that tumor progression may significantly depend on alterations in cancer-associated stromal cell signaling.^5^ However, no attempt has been made to integrate stromal cells into the prognostic prediction model for uterine corpus endometrioid cancer (UCEC) to improve accuracy. In this study, we successfully identified stromal cell differentiation fate genes (SDFGs) and developed a novel UCEC risk stratification system based on these genes.

Firstly, we performed t-SNE dimensionality reduction analysis on the single-cell RNA sequencing data of 35,283 cells from five UCEC tissues of GSE173682. Thirteen clusters were identified, and six cell types, including B cells, endothelial cells, epithelial cells, myeloid cells, natural killer/T cells, and stromal cells, were annotated (Fig. 1A; Fig. S1A–D). Next, we extracted stromal cells and divided these cells into eight clusters (Fig. 1B; Fig. S1E, F). To identify stromal cell fate-related genes, we performed Monocle 2 analysis and uncovered five distinct differentiation states as well as 1130 genes significant in Monocle 2 analysis of stromal cells (Fig. 1C; Fig. S1G). Intersecting with 116 significant genes in the Kaplan–Meier survival analysis and 39 significant genes in the univariate Cox regression analysis of the TCGA-UCEC cohort, we finally defined 37 SDFGs (Table S1 and Fig. 1D).

**Figure 1:**
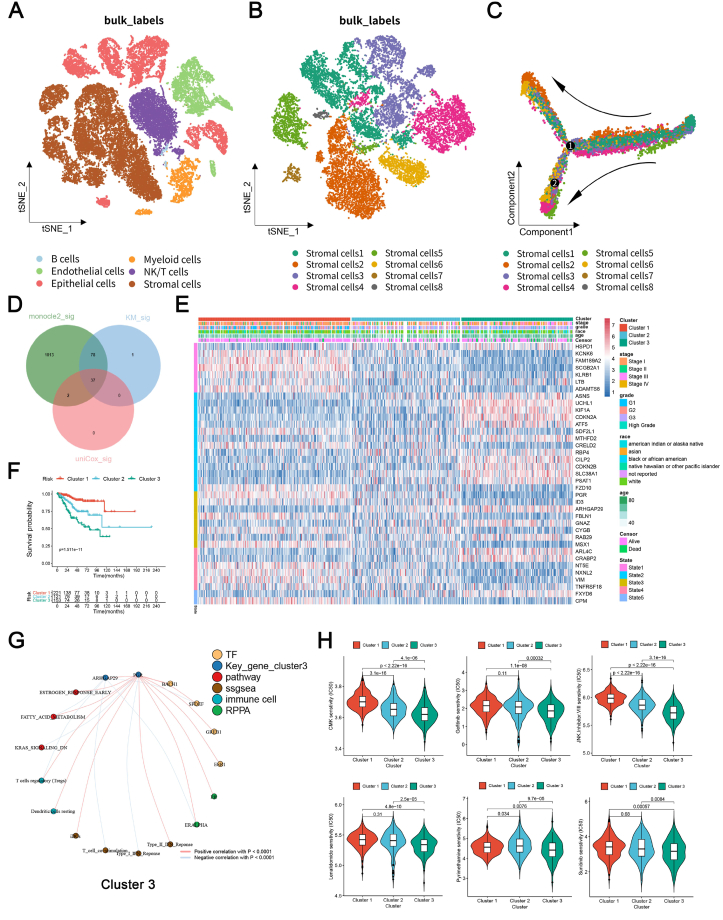
Identification of stromal cell fates and construction of a novel risk stratification system in UCEC. **(A)** t-SNE plots exhibited 35,283 cells, with six cell types identified (B cells, endothelial cells, epithelial cells, myeloid cells, NK/T cells, and stromal cells) in UCEC. **(B)** Eight subpopulations within 15,582 stromal cells were identified in UCEC. **(C)** The pseudo-temporal trajectory of stromal cells, with all subpopulations annotated. The arrows indicate the direction of the inferred differentiation trajectories. **(D)** Thirty-seven SDFGs were identified through Monocle 2 analysis, Kaplan–Meier survival analysis, and univariate Cox regression analysis. **(E)** The heatmap of the three clusters based on consensus clustering analysis. **(F)** Different survival probabilities of three clusters were revealed by the Kaplan–Meier survival analysis. **(G)** A separate co-expression regulatory network for cluster 3 was constructed to characterize its unique functions, demonstrating tight regulatory relationships between various components. **(H)** Possible target drugs with the lowest IC_50_ for cluster 3 predicted based on the pRRophetic algorithm.

Based on 37 SDFGs, we performed consensus clustering of TCGA bulk RNA sequencing data from UCEC patients to establish a UCEC risk stratification system consisting of three clusters. The differential expression of all 37 SDFGs across various clusters was illustrated in Figure S2A. The heatmap revealed differential expression of 37 SDFGs across five states, showing that determinant genes for states 1 and 2 were highly expressed in clusters 1 and 3, respectively (Fig. 1E). The Kaplan–Meier survival curves for the three clusters also revealed significant differences (*p* < 0.001), with cluster 1 harboring the best prognosis and cluster 3 harboring the worst (Fig. 1F). Next, we displayed significant discrimination between the three clusters by principal component analysis (PCA) (Fig. S2B). The high PCA score group had a significantly worse prognosis than the low PCA score group (*p* < 0.001; Fig. S2C), with cluster 3 showing the highest scores and poorest prognosis and cluster 1 showing the lowest scores and the best prognosis (*p* < 0.001; Fig. S2D). Subsequently, we investigated the multi-omics correlations of the UCEC risk stratification system. “High PCA score plus high tumor mutation burden” was associated with the lowest survival probability (Fig. S2E). Cluster 3, showing the highest PCA scores, was negatively correlated with most immune cell infiltration (Fig. S2F, G) and was predicted to be not responsive to immune checkpoint inhibitors (Fig. S2H).

Moreover, we constructed a prognostic prediction model based on SDFGs. To prevent overfitting, we applied LASSO regression, and 16 genes were selected to construct the prognostic prediction model (Fig. S3A, B). We then divided the UCEC patients into the training and test groups at a 7-to-3 ratio and further separated them into the low- and high-risk groups based on the median risk scores. The differential expression of the 16 genes between the high- and low-risk groups was shown in Figure S3C. According to the receiver operating characteristic (ROC) curves, the area under the ROC curve (AUC) in the all set, training set, and testing set reached 0.800, 0.812, and 0.772, respectively, displaying the credibility of the model (Fig. S3D). To determine whether the risk score was an independent prognostic factor, we incorporated “age” “grade” “bone metastasis” “distant metastasis” “stage” and “risk score” into univariate and multivariate Cox regression analyses, and found that the risk score was an independent risk factor of the UCEC patients' prognosis (hazard ratio = 21.94, *p* < 0.001, and hazard ratio = 8.89, *p* < 0.001, respectively) (Fig. S3E). Ultimately, we intended to discover the potential mechanisms of each consensus cluster. We first conducted differential expression analysis between high-stage (III/IV) and low-stage (I/II) UCEC patients and discovered several differentially expressed SDFGs, transcription factors, and hallmark pathways. Based on these components, we constructed the co-expression regulatory network for each cluster (Fig. 1G; Fig. S4A, C). ARHGAP29 and PGR were identified as key genes for cluster 3, which harbored the worst prognosis. The possible target drugs for each cluster are listed in Figure 1H and Figure S4B and D.

All in all, our study introduced a novel UCEC risk stratification system based on SDFGs with multi-omics correlations, highlighting the critical role of stromal cells in UCEC progression. The identification of 37 SDFGs and their integration into the risk model enhanced classification and prognostic assessment. Most importantly, we identified two key genes (ARHGAP29 and PGR) in the co-regulatory network of cluster 3, with the worst prognosis, as well as the potential target drugs. Further study may focus on these key genes and potential drugs and try to reveal their possible regulatory mechanisms. In clinical practice, the detection of the key genes identified in our UCEC risk stratification system might help in the early diagnosis of those high-risk UCEC patients and provide opportunities for early treatment.

## CRediT authorship contribution statement

**Rulin Zhang:** Writing – review & editing, Writing – original draft, Formal analysis, Data curation. **Haonan Ma:** Writing – review & editing, Writing – original draft, Formal analysis, Data curation. **Yiting Yang:** Writing – review & editing, Writing – original draft, Formal analysis, Data curation. **Siang Lv:** Writing – review & editing, Data curation. **Xin Guan:** Writing – review & editing, Data curation. **Bingnan Lu:** Writing – review & editing, Data curation. **Yuntao Yao:** Writing – review & editing, Data curation. **Runzhi Huang:** Writing – review & editing, Formal analysis. **Yifan Liu:** Writing – review & editing, Writing – original draft, Formal analysis. **Yanhua Du:** Writing – review & editing, Writing – original draft. **Jun Wu:** Writing – review & editing, Writing – original draft, Funding acquisition.

## Data accessibility

The datasets generated and/or analyzed during the study are available in the GEO database (https://www.ncbi.nlm.nih.gov/geo/), TCGA database (https://tcga-data.nci.nih.gov/tcga/), Cistrome Cancer database (http://cistrome.org/), and MSigDB database (https://www.gsea-msigdb.org/gsea/msigdb/index.jsp).

## Funding

This work was sponsored in part by the Natural Science Foundation of Guangxi, China (No.2023GXNSFAA026276), the 10.13039/501100001809National Natural Science Foundation of China (No. 82072892), the Key Discipline Construction Project of Jiading District Health System (Shanghai, China) (No. XK202405) and the Research Innovation Project of Jiading Branch of Shanghai General Hospital, China (No. 2025-KJ-PD-01). The funders had no role in study design, data collection and analysis, the decision to publish, or the preparation of the manuscript.

## Conflict of interests

The authors declared no conflict of interests.
