# Supplementary material for: Uncovering stromal cell fate genes and a novel risk stratification in UCEC by integrating single-cell RNA sequencing and multi-omics analysis
Source: Genes Dis. 2025 Jun 27;13(1):101743. doi: 10.1016/j.gendis.2025.101743 (PMC12466133; doi:10.1016/j.gendis.2025.101743)
Supplement: Multimedia component 1 [file mmc1.pdf]

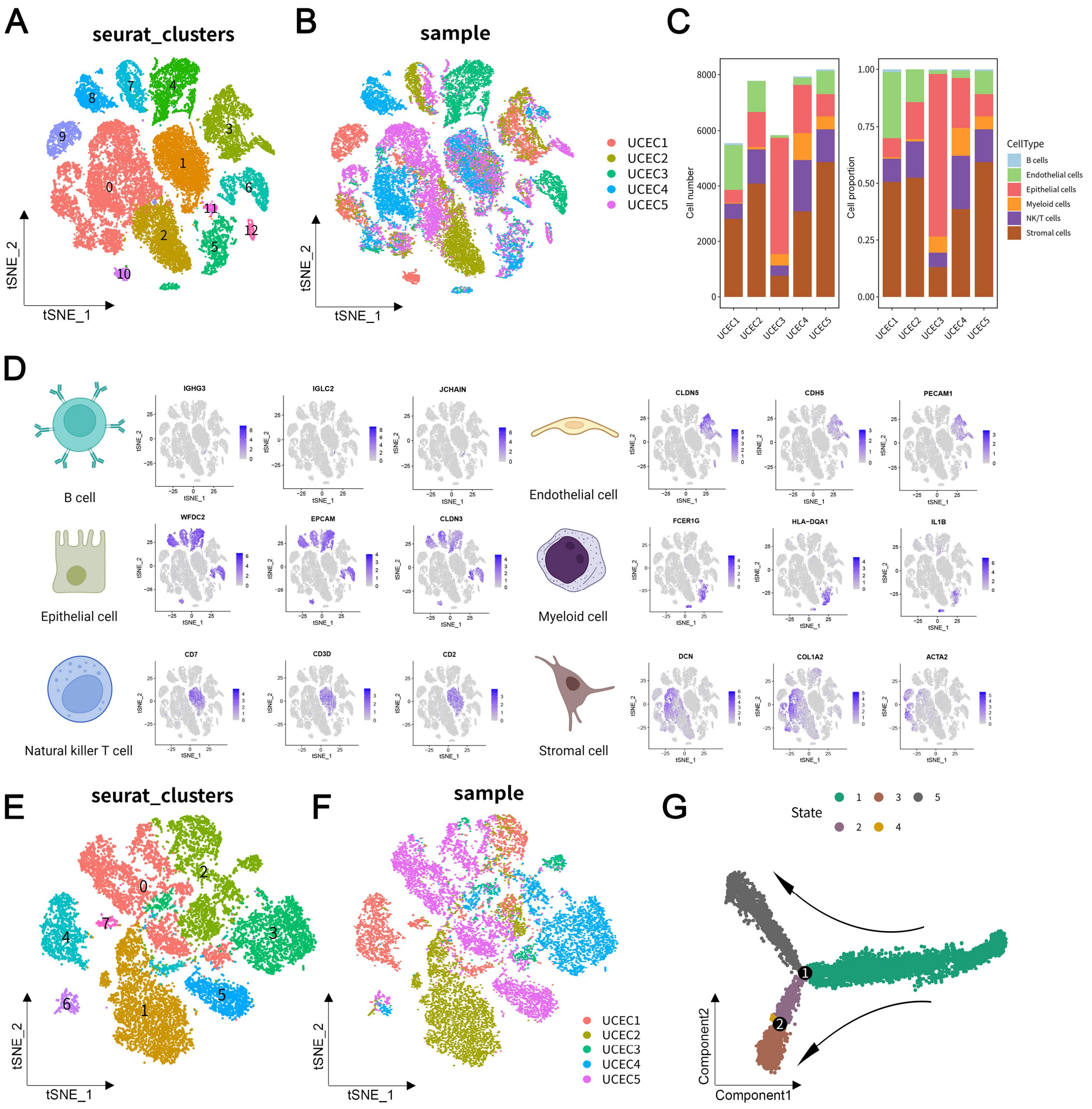

Figure S1. Characteristics of UCEC and stromal cells in UCEC.

(A) 13 seurat clusters were identified in UCEC.

(B) The belonging relations between each cluster and five UCEC samples were shown in the t-SNE plot.

(C) The bar graphs represented the number and percentage of each cell type in all samples.

(D) The t-SNE feature plots exhibited the characteristic plots of marker gene expression for each cell type.

(E) Eight seurat clusters were identified in stromal cells in UCEC.

(F) The belonging relations between each stromal cell cluster and five UCEC samples were shown in the t-SNE plot.

(G) Pseudo-temporal trajectory of stromal cells, with all states annotated was shown in the plot.

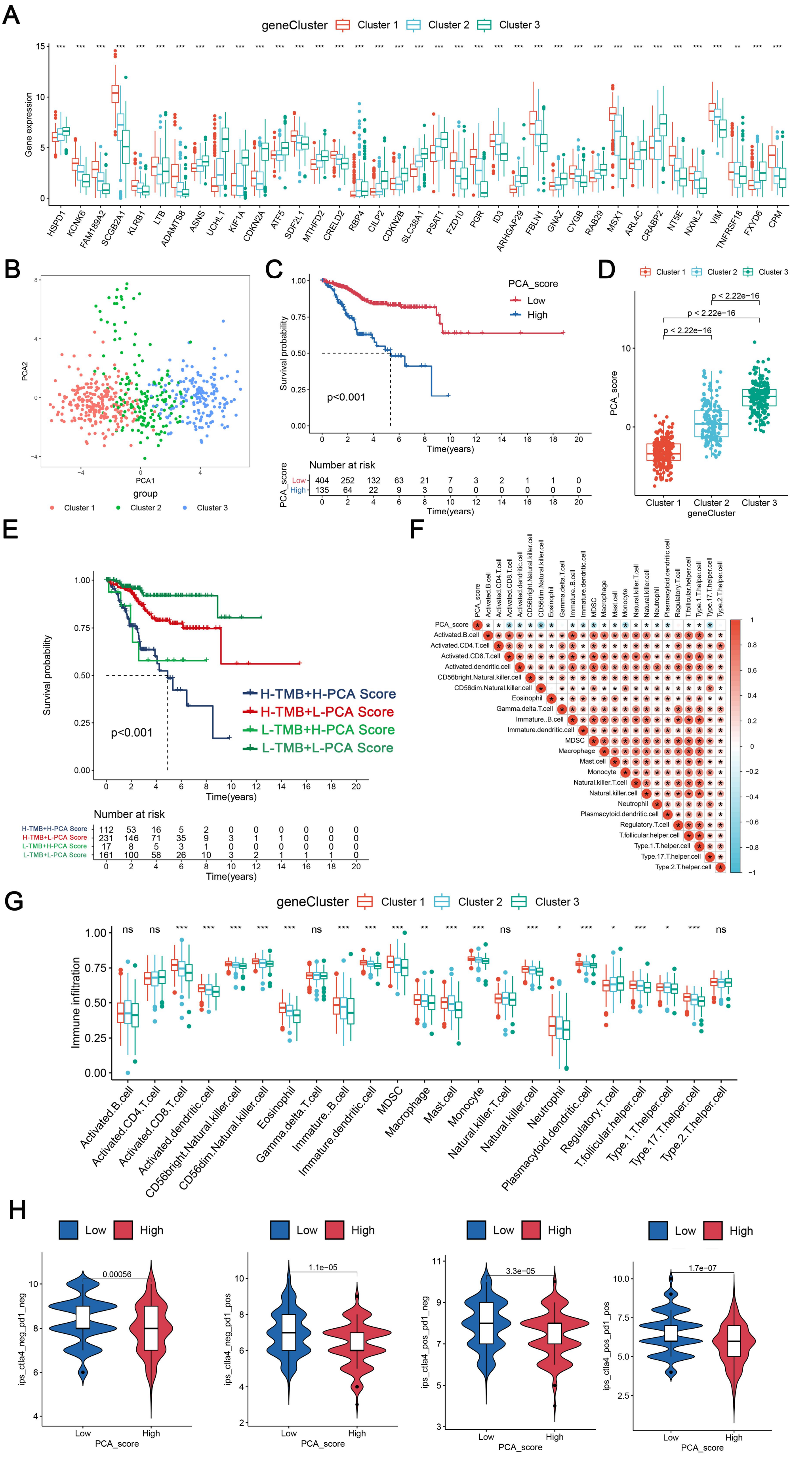
A
geneCluster Cluster 1 Cluster 2 Cluster 3
Gene expression
HSPD1 KCNK6 FAM189A2 SCGB2A1 KLRB1 LTB ADAMTS8 ASNS UCHL1 KIF1A CDKN2A ATF5 SDF2L1 MTHFD2 CRELD2 RBP4 CILP2 CDKN2B SLC38A1 PSAT1 FZD10 PGR ID3 ARHGAP29 FBLN1 GNAZ CYGB RAB29 MSX1 ARL4C CRABP2 NT5E NXNL2 VIM TNFRSF18 FXYD6 CPM
B
PCA2
PCA1
group
Cluster 1 Cluster 2 Cluster 3
C
PCA_score
Low
High
Survival probability
p<0.001
Time(years)
Number at risk
Low 404 252 132 63 21 7 3 2 1 1 0
High 135 64 22 9 3 0 0 0 0 0 0
D
Cluster 1 Cluster 2 Cluster 3
p < 2.22e−16
p < 2.22e−16
p < 2.22e−16
PCA_score
geneCluster
E
Survival probability
H-TMB+H-PCA Score
H-TMB+L-PCA Score
L-TMB+H-PCA Score
L-TMB+L-PCA Score
p<0.001
Time(years)
Number at risk
H-TMB+H-PCA Score 112 53 16 5 2 0 0 0 0 0 0
H-TMB+L-PCA Score 231 146 71 35 9 3 1 1 0 0 0
L-TMB+H-PCA Score 17 8 5 3 1 0 0 0 0 0 0
L-TMB+L-PCA Score 161 100 58 26 10 3 2 1 1 1 0
F
PCA_score
Activated.B.cell
Activated.CD4.T.cell
Activated.CD8.T.cell
Activated.dendritic.cell
CD56bright.Natural.killer.cell
CD56dim.Natural.killer.cell
Eosinophil
Gamma.delta.T.cell
Immature..B.cell
Immature.dendritic.cell
MDSC
Macrophage
Mast.cell
Monocyte
Natural.killer.T.cell
Natural.killer.cell
Neutrophil
Plasmacytoid.dendritic.cell
Regulatory.T.cell
T.follicular.helper.cell
Type.1.T.helper.cell
Type.17.T.helper.cell
Type.2.T.helper.cell
G
geneCluster Cluster 1 Cluster 2 Cluster 3
Immune infiltration
Activated.B.cell Activated.CD4.T.cell Activated.CD8.T.cell Activated.dendritic.cell CD56bright.Natural.killer.cell CD56dim.Natural.killer.cell Eosinophil Gamma.delta.T.cell Immature..B.cell Immature.dendritic.cell MDSC Macrophage Mast.cell Monocyte Natural.killer.T.cell Natural.killer.cell Neutrophil Plasmacytoid.dendritic.cell Regulatory.T.cell T.follicular.helper.cell Type.1.T.helper.cell Type.17.T.helper.cell Type.2.T.helper.cell
H
Low High
0.00056
ips_ctla4_neg_pd1_neg
1.1e−05
ips_ctla4_neg_pd1_pos
3.3e−05
ips_ctla4_pos_pd1_neg
1.7e−07
ips_ctla4_pos_pd1_pos
PCA_score

Figure S2. Multi-omics correlations of the UCEC risk stratification system.

(A) The expression levels of SDFGs for three clusters were shown in the boxplot.

(B) Three clusters were located away from each other in the PCA plot.

(C) Patients with high PCA scores were usually associated with worse clinical prognosis.

(D) The corresponding relationships between three clusters and PCA scores were illustrated.

(E) The survival probability of “PCA score” and “TMB” subgroups were shown. Patients with “High PCA score + high TMB” harbored the lowest survival probability.

(F) A correlation matrix of 23 types of immune cells and PCA score were exhibited. PCA score was negatively correlated with most immune cell infiltration

(G) The boxplot exhibited the immune cell infiltration of each cluster.

(H) The differences of immune checkpoint inhibitor response in high- and low-PCA-score subgroups were exhibited.

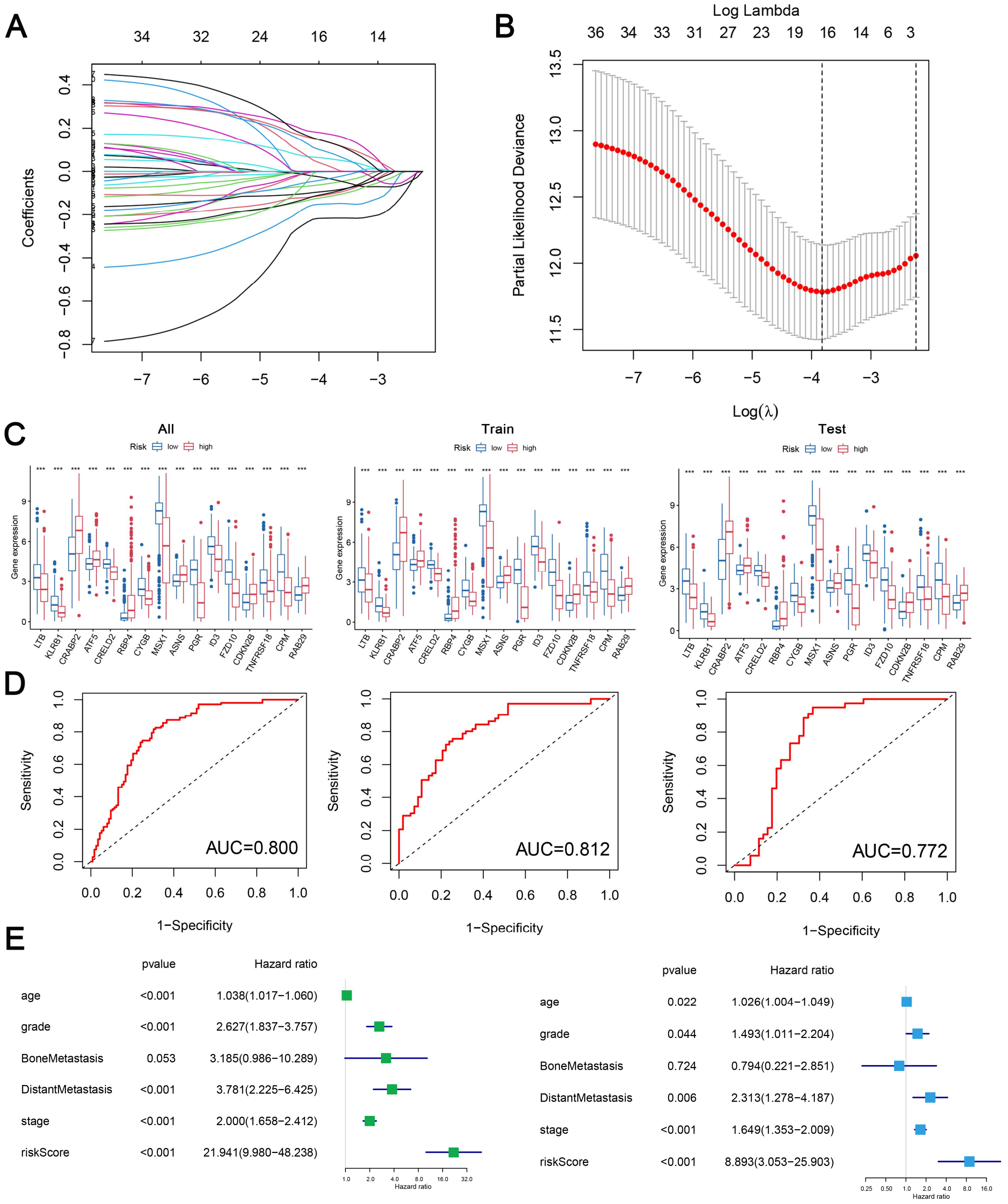

Figure S3. Construction of the prognostic model based on SDFGs.

(A) (B) Lasso regression determined the total number of SDFGs incorporated in the model.

(C) Boxplots showed significant expression differences of 16 genes between the high- and low-risk groups.

(D) The AUC value in the all set, training set, and testing set reached 0.800, 0.812, and 0.772, respectively

(E) The risk score was an independent risk factor of the UCEC patients' prognosis (HR = 21.94, P < 0.001 (univariate Cox regression analysis, left), and HR = 8.89, P < 0.001 (multivariate Cox regression analysis, right))

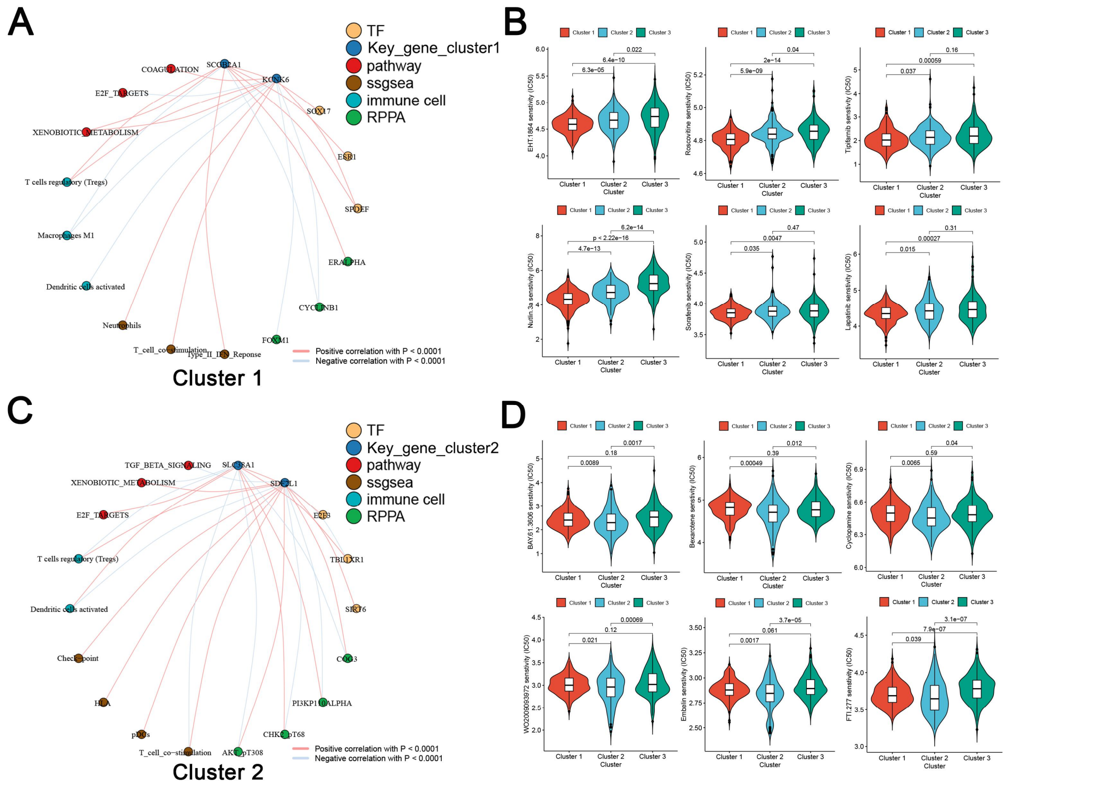

Figure S4. Construction of co-expression regulatory networks and prediction of target drugs.

(A) The separate co-expression regulatory network for cluster 1 was exhibited.

(B) Possible target drugs with the lowest IC50 for cluster 1 were exhibited.

(C) The separate co-expression regulatory network for cluster 2 was exhibited.

(D) Possible target drugs with the lowest IC50 for cluster 2 were exhibited.

TABLE S1 | The final stromal cell differentiation fate genes (SDFGs)

Id	HR	HR.95L	HR.95H	Cox_pvalue	KM_pvalue
ARL4C	1.369	1.159	1.618	0.000	0.000
HSPD1	1.624	1.220	2.161	0.001	0.000
PGR	0.739	0.651	0.839	0.000	0.000
ID3	0.763	0.643	0.906	0.002	0.000
ASNS	1.469	1.145	1.884	0.002	0.000
CRABP2	1.231	1.106	1.370	0.000	0.000

UCHL1	1.174	1.081	1.276	0.000	0.000
NT5E	0.809	0.707	0.925	0.002	0.001
KIF1A	1.249	1.109	1.407	0.000	0.000
ARHGAP29	1.420	1.170	1.724	0.000	0.000
CDKN2A	1.285	1.151	1.434	0.000	0.000
ATF5	1.418	1.147	1.754	0.001	0.000
FBLN1	0.834	0.745	0.933	0.001	0.000
KCNK6	0.627	0.512	0.768	0.000	0.000
SDF2L1	0.729	0.596	0.893	0.002	0.000
MTHFD2	1.540	1.223	1.938	0.000	0.000
FAM189A2	0.685	0.567	0.828	0.000	0.000
CRELD2	0.543	0.420	0.702	0.000	0.000
GNAZ	1.458	1.141	1.864	0.003	0.000
RBP4	1.179	1.060	1.310	0.002	0.000
CYGB	0.655	0.512	0.837	0.001	0.000
CILP2	1.327	1.110	1.585	0.002	0.000
CDKN2B	1.496	1.240	1.806	0.000	0.000
NXNL2	0.683	0.555	0.840	0.000	0.000
SLC38A1	1.431	1.185	1.727	0.000	0.000
SCGB2A1	0.865	0.813	0.921	0.000	0.000
VIM	0.752	0.649	0.871	0.000	0.000
TNFRSF18	0.769	0.653	0.906	0.002	0.000
RAB29	1.533	1.201	1.957	0.001	0.000
KLRB1	0.511	0.360	0.725	0.000	0.000
LTB	0.777	0.667	0.904	0.001	0.000
ADAMTS8	0.733	0.600	0.895	0.002	0.000
PSAT1	1.326	1.127	1.560	0.001	0.000
CPM	0.799	0.696	0.917	0.001	0.000
FZD10	0.799	0.696	0.918	0.001	0.000
MSX1	0.866	0.806	0.929	0.000	0.000
FXYD6	1.263	1.082	1.473	0.003	0.000
