# Supplementary material for: Uncovering stromal cell fate genes and a novel risk stratification in UCEC by integrating single-cell RNA sequencing and multi-omics analysis
Source: Genes Dis. 2025 Jun 27;13(1):101743. doi: 10.1016/j.gendis.2025.101743 (PMC12466133; doi:10.1016/j.gendis.2025.101743)
Supplement: Multimedia component 2 [file mmc2.docx]

**Methods**

**Data sources**

The single-cell RNA sequencing data (scRNA-seq) of UCEC samples was extracted from the Gene Expression Omnibus (GEO) database ([http://www.ncbi.nlm.nih.gov/geo/](https://www.ncbi.nlm.nih.gov/geo/)) (GEO accession: GSE173682)^[1]^. A total of 35,283 cells from UCEC patients in this dataset were included in the study. We downloaded the bulk RNA-seq and clinical data of UCEC samples from the TCGA database ([https://tcga-data.nci.nih.gov/tcga/](https://www.cancer.gov/ccg/research/genome-sequencing/tcga))^[2]^, which contains survival data for 539 samples.

**Primary scRNA-seq data processing, DEG Identification and cell annotation**

We used 10x Genomics Chromium System (<https://www.10xgenomics.com/instruments/chromium-x-series>) to preprocess the data. Cellranger mkfastq from the Cell Ranger software package (v3.0, [http://10xgenomics.com/](https://www.10xgenomics.com/)) was applied to create fastq files from raw scRNA-seq data. Demultiplexed fastq data were aligned to the GRCm38 reference transcriptome, and then the count of gene expression matrix was taken employing the cellranger count software. Then, we applied the Seurat pipeline (version 3.2.2,<https://satijalab.org/seurat/>) to further analyze the quantitative gene expression matrix. We excluded genes that were detected in less than three cells and also cells with less than 100,000 transcripts and limited the mitochondria proportion to less than 10%. Then we used the "IntegrateData" function to merge the contained samples into one Seurat object which was normalized and scaled using the "ScaleData" function. Using "vst" selection, the top 2,000 variable genes were selected which were then used as the input features for initial dimensionality reduction with principal component analysis (PCA). After calculating a shared nearest-neighbor graph with the Louvain algorithm embedded in the "FindClusters" function, the clustering analysis was carried out via the integrated joint embedding generated by Harmony with the Louvain algorithm. The 2D map created using the t-Distributed Stochastic Neighbor Embedding (t-SNE) method displayed the identified clusters. Subsequently, we identified differentially expressed genes (DEGs) through the “FindAllMarkers” function with default parameters with |log2(fold change)| > 0.5 (Wilcoxon rank-sum test) and adjusted p-value < 0.05 (Bonferroni correction). Using the CellMarker database as a reference, the gene expression of well-defined cell markers was examined in order to identify the distinct cell type of each unregulated cluster^[3]^. We then retained only a total of 15,582 stromal cells for separate analysis which repeated the above steps.

**Trajectory analysis**

The pseudotime trajectories of stromal cells were determined using the Monocle 2 R package (version 2.8.0)^[4]^. First, we used the differential GeneTest function to extract the ordering genes from the DEGs between distinct stromal cell clusters, and then use the reversed graph embedding algorithm to sort the single cells on a low-dimensional space reduced by expression profiles, thus forming a pseudo-temporal trajectory.

**Identification of final stromal cell fate gene set**

We performed univariate Cox regression and Kaplan-Meier (K-M) analyses on the bulk RNA-seq data and the survival data of UCEC patients from TCGA database. Then we entered the genes significant in Monocle2, Cox regression, and K-M analysis into jvenn online mapping software (<http://www.bioinformatics.com.cn/static/others/jvenn/example.html>)^[5]^, and the intersection set obtained was the final stromal cell fate gene set, which would be used for the next step of clustering analysis.

**Consensus clustering**

Using ConsensusClusterPlus clustering analysis, UCEC patients in the TCGA database were divided into distinct groups according to the expression levels of the stromal cell fate genes described above, and clustering labels for different differentiation states were obtained^[6,7]^. We obtained PCA scores using Principal Component Analysis^[8]^, and later performed survival analysis and multi-omics analysis based on PCA scores.

**Multi-omics correlation analysis**

We explored multi-omics correlations of clustering labels and PCA scores. We combined the PCA scores and tumor mutation burden (TMB) scores of different grades separately and performed K-M survival analysis. Single sample gene set enrichment analysis (ssGSEA) was used to make predictions about the levels of infiltration of 23 immune cell subgroups involved in innate and adaptive immunity^[9]^. We also evaluated the immunotherapy sensitivities by assessing the drug responses of CTLA-4 and PD-1 (The Cancer Immunome Atlas (TCIA) database (https://www.tcia.at/)).

**Validation of the stromal cell fate gene set's predictive values**

Stromal cell fate genes were screened using lasso regression to avoid overfitting, and 16 genes were eventually integrated into a multifactorial Cox prognostic model whose accuracy was assessed by Receiver Operating Characteristic (ROC) curves. The following formula was used to calculate the risk score (RS) for each UCEC sample:

Risk score = β1×fate gene 1+β2×fate gene 2+⋯+βn×fate gene n

In this formula, "β" represents the coefficient of each stromal cell fate gene, and "fate gene n" represents the expression level of the n^th^ fate gene. After that, patients were divided into low-risk and high-risk groups based on the median risk score of all patients. Additionally, we conducted univariate and multivariate Cox regression analysis once again, integrating demographic data for model correction, such as age, gender, to evaluate the independent prognostic value of RS.

**Construction of regulatory networks**

We employed Pearson correlation analysis to identify significant correlations between stromal cell fate genes and TFs, RPPA proteins, as well as hallmark signaling pathways, immune cells, enriched immune gene sets. The relationships of these components for each cluster were visualized in co-expression regulatory networks

**Inhibitor Prediction**

To predict the sensitivity of each group of patients to different chemotherapeutic agents, we calculated the half-maximal inhibitory concentration (IC50) using the pRRophetic R package^[10]^. The results were visualized in violin plots.

#### References

[1] Regner M J, Wisniewska K, Garcia-Recio S, et al.A multi-omic single-cell landscape of human gynecologic malignancies[J].Mol Cell,2021, 81 (23): 4924-4941 e10.

[2] Cancer Genome Atlas Research N, Weinstein J N, Collisson E A, et al.The Cancer Genome

[3] Zhang X, Lan Y, Xu J, et al.CellMarker: a manually curated resource of cell markers in human and mouse[J].Nucleic Acids Res,2019, 47 (D1): D721-D728.

[4] Qiu X, Mao Q, Tang Y, et al.Reversed graph embedding resolves complex single-cell trajectories[J].Nat Methods,2017, 14 (10): 979-982.

[5] Bardou P, Mariette J, Escudie F, et al.jvenn: an interactive Venn diagram viewer[J].BMC Bioinformatics,2014, 15 (1): 293.

[6] Wilkerson M D, Hayes D N.ConsensusClusterPlus: a class discovery tool with confidence assessments and item tracking[J].Bioinformatics,2010, 26 (12): 1572-3.

[7] Reich M, Liefeld T, Gould J, et al.GenePattern 2.0[J].Nat Genet,2006, 38 (5): 500-1.

[8] Ringner M.What is principal component analysis?[J].Nat Biotechnol,2008, 26 (3): 303-4.

[9] Newman A M, Steen C B, Liu C L, et al.Determining cell type abundance and expression from bulk tissues with digital cytometry[J].Nat Biotechnol,2019, 37 (7): 773-782.

[10] He Y, Jiang Z, Chen C, et al.Classification of triple-negative breast cancers based on Immunogenomic profiling[J].J Exp Clin Cancer Res,2018, 37 (1): 327.
